# The use of teleorthodontics during the COVID-19 pandemic and beyond – perspectives of patients and providers

**DOI:** 10.1186/s12903-023-03215-4

**Published:** 2023-07-15

**Authors:** Karen Homsi, Vinitha Ramachandran, Dhammacari Martin Del Campo, Laura Martin Del Campo, Budi Kusnoto, Phimon Atsawasuwan, Grace Viana, Maysaa Oubaidin, Veerasathpurush Allareddy, Mohammed H. Elnagar

**Affiliations:** Department of Orthodontics, College of Dentistry, University of Illinois Chicago, Chicago, IL USA

**Keywords:** COVID-19, Teledentistry, Teleorthodontics, Distant Dental care

## Abstract

**Background:**

The COVID-19 pandemic significantly impacted dental services, resulting in reduced staff availability, limited appointments, and some dental clinics even being forced to close their doors. Despite these challenges, the need for dental consultants remained present, particularly in emergency situations. One area of orthodontics that had seen a surge in demand during the pandemic is Teleorthodontics. With the help of Teleorthodontics, orthodontic consultations, assessments, and even treatment monitoring could be conducted remotely, making it a safe and convenient option for patients during those challenging times.

**Aim:**

This survey aimed to evaluate the acceptance of patients and their orthodontists on the use of different modes of communication through Teleorthodontics during the COVID-19 pandemic and their willingness to continue using this in the future.

**Methods:**

An online survey instrument in Qualtrics was distributed to orthodontic patients at the University of Illinois, Chicago. The survey was available on a rolling basis for up to 6 months. A total number of 364 partients voluntarily participated in the survey. The Faculty and Residents were also asked to participate in a survey through recruitment via their UIC email addresses.

**Results:**

According to our survey, both patients and providers showed acceptance of Teleorthodontics and have used it in different forms during orthodontic treatment. The application is easy-to-use, convenient, and not at all time-consuming. Overall satisfaction with using this application was recorded at 92%, with 66% of patients stating that it saved them time by eliminating the need to travel to the orthodontic clinic. 30% of providers found that the interaction with patients using Teleorthodontics was a positive experience and would recommend it in future.

**Conclusion:**

Teleorthodontics has shown great potential, particularly in follow-up cases, and holds promise as a valuable tool for online remote dental consultations in the future.

## Background

The COVID pandemic forced the world to adapt to a new way of life where social distancing and staying at home became the norm.

Due to the high virulence and transmissibility of this virus, regular activities were halted. The risk of infection was particularly high for dental professionals who work closely with patients, as transmission through droplets of saliva while coughing, sneezing, or even talking was possible. The use of handpiece drilling instruments, which emit tiny aerosol particles, also posed a significant risk [1]. Droplets containing infectious particles can stay suspended in the air for a while before settling on surfaces, potentially creating an infectious environment [2]. Unfortunately, these droplets can also infect other surfaces, making them another means of transmission. Due to this risk, many dental care facilities had closed completely to suspend all dental procedures and prevent the spread of infection. This, combined with limited access to general hospitals, made it challenging for many people to access the care they needed. Approximately 20% of the American population lives in rural areas, making it even harder for them to access dental services during the pandemic. Clinical examination has traditionally been the standard method for diagnosing dental problems and recommending treatment. However, due to the pandemic, dental care providers had to adapt to new protocols in order to continue providing essential services. In order to provide uninterrupted dental care services to patients, it became necessary to explore other modes of communication. The latest advancements in digital technology made this possible, leading to the development of Teledentistry. This growing field uses technology to deliver dental care to patients in remote locations and across geographic regions, and has significantly transformed dental practice. In the event of another pandemic or for other containment reasons, Teledentistry will also be of interest in this uncertain world. The term “*Teledentistry*” was first used by Cook in 1997, which he defined as “*the practice of using video-conferencing technologies to diagnose and provide advice about treatment over a distance*” [3].

Teledentistry offers various modes that provide patients with quick, easy, and convenient ways to consult with dental professionals. Using clinical photographs and radiographic images shared by patients from remote areas, diagnosis and treatment planning can be easily conducted. Moreover, electronic prescriptions for pain or infections, as well as follow-up or review of certain cases, can all be swiftly and conveniently done through Teledentistry.

According to recent studies, teledentistry has been proven to enhance oral hygiene and improve patient compliance, leading to better treatment outcomes [4].

One major advantage of Teledentistry is that it can help to minimize the spread of infections, which is typically a major concern in traditional dental offices. The urgent need for patients to contact dental consultants during certain dental emergencies resulted in an increase of emails and texts to their concerned dentists. This led to a wider use and adoption of Teledentistry, which was initially more prevalent in emergency cases, but eventually replaced traditional in-person consultations. It was particularly useful for patients who lived in remote areas. Different fields of dentistry adopted this mode of communication with patients during this time including Orthodontics, thus leading to the birth of Teleorthodontics. Teleorthodontics is an innovative approach to orthodontic care that utilizes digital and communication technologies to provide remote consultations, diagnosis, and monitoring of patients’ treatment progress. With the help of Teleorthodontics, patients can consult with their orthodontist from the comfort of their own homes, without the need for in-person visits. This technology also allows for real-time communication between patients and orthodontists, enabling them to address any concerns or changes in treatment plans promptly. Additionally, Teleorthodontics has proven to be a convenient solution for patients who may have mobility issues or live in remote areas without access to specialized care.

This is a survey-based study aimed to conduct a descriptive and exploratory analysis of the facilitators and barriers to providing orthodontic care across different modes of virtual communication platforms over an extended period of no onsite visits during the COVID-19 pandemic by way of,

1. Evaluation of patient feedback and satisfaction.
2. Evaluation of the Provider’s /orthodontist’s feedback.

This study seeks to focus on the following platforms: phone-voice only, exchange of text only, exchange of text and multimedia, live video and sound, and Dental Monitoring TM (DM), which can be used to send text messages and video consultations through the specific application. The asynchronous platforms include the exchange of information through SMS text and email. Through these platforms, patients can ask questions, take pictures of their teeth and send them to their provider at any point in time. Live video conferencing and voice-only phone interactions will be regarded as synchronous forms of telecommunications.

## Methods

A voluntary and anonymous cross-sectional survey of patients (responders) and orthodontists (providers) was conducted using the user-friendly survey software UIC Qualtrics.

An online survey instrument in Qualtrics format was distributed via fliers with HTML and QR code links posted at the University of Illinois Chicago, College of Dentistry (UIC COD) Department of Orthodontics clinic. The survey was available on a rolling basis for up to 6 months with the consent form when logging in. After signing consent forms, survey participants were asked to answer questions regarding demographics, their association with the UIC COD Department of Orthodontics, and usage and preferences regarding Teleorthodontic communication. After completing the survey, subjects received compensation in the form of $5 cash.

The faculty and residents were recruited to participate in a survey via their official email addresses.

The initial questions encountered helped further determine eligibility. Patients who responded to questions demonstrating a lack of eligibility were redirected to the end of the survey and did not have the opportunity to progress further through the survey instrument.

### Participants were recruited according to the following -

#### Inclusion criteria

Patient participants:

Must be a patient (or parent/guardian of a patient) of the UIC COD Department of Orthodontics., Must be over 18 years of age.

Provider participants: Must be a UIC COD Department of Orthodontics resident or faculty member.

#### Exclusion criteria

Unable to read and understand English or Spanish.

Has already participated in this survey research study.

**Fig. 1:**
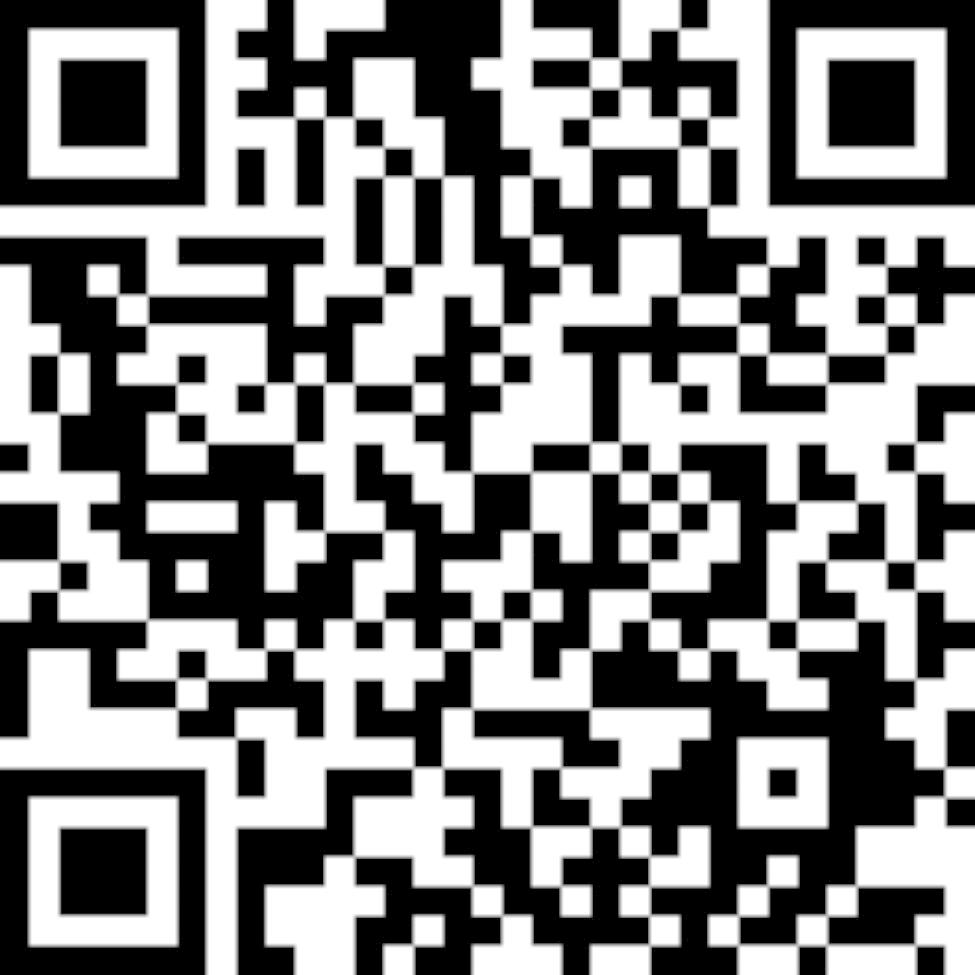
QR code for questionnaire distributed to patients

**Fig. 2:**
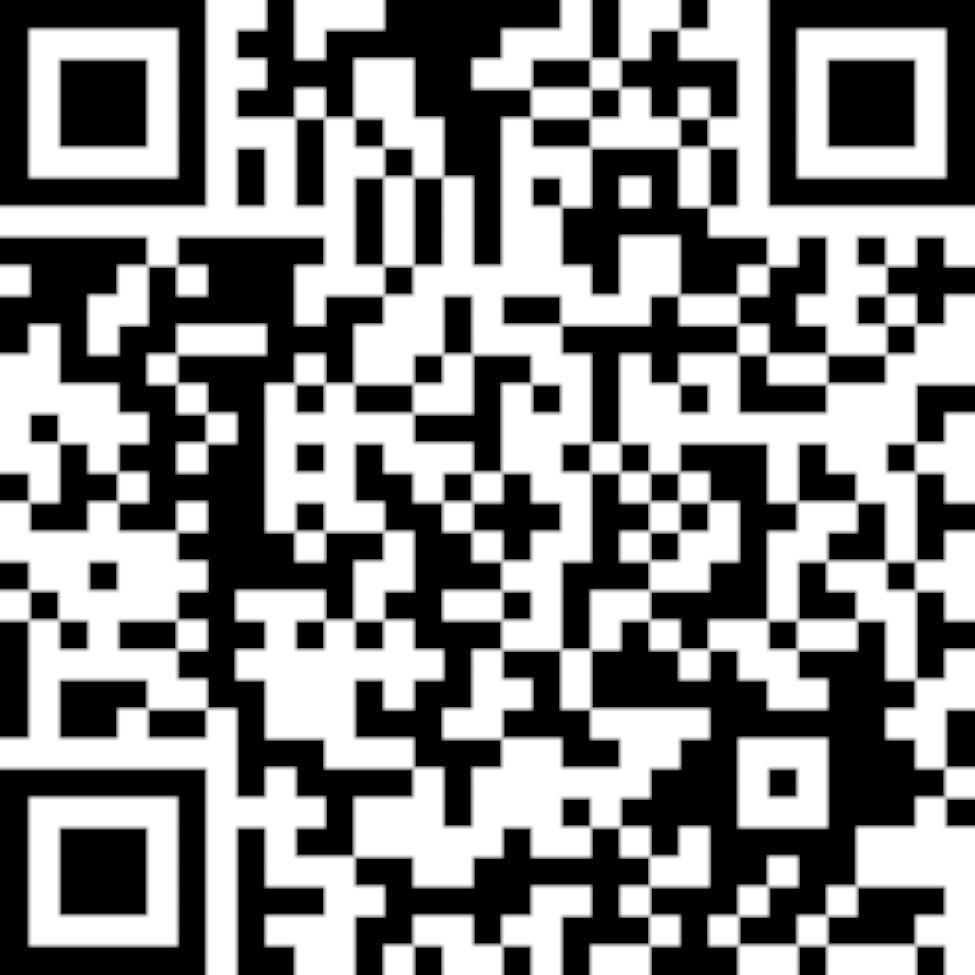
QR code for questionnaire distributed to providers

The following Figures show the QR codes which lead to the Questionnaires distributed to the patients (Fig. 1.) and Providers/orthodontists (Fig. 2.)

### Electronic Qualtrics Survey Instrument

The survey instrument was available in both English and Spanish for patients and a separate survey in English for providers.Missing or incomplete information was used to the degree possible in the data analysis. The survey was made available in December 2021.

A Descriptive Analysis using the distribution of frequencies (%), was done based on the survey responses of the patients and providers. The data was analyzed using IBM SPSS Statistics for Windows, version 28.0.

## Results

The Teleorthodontic application was introduced during the period from December 2021 to May 2022 during the COVID-19 outbreak.

### Study participants’ characteristics

Three hundred and sixty-four patients with the age group range from around 18–65 years of age participated voluntarily in the survey. 57.7% of the patient participants were a parent or legal guardian of a patient being treated at the orthodontic clinic, while 41.4% were actual orthodontic patients treated on a regular basis. (Shown in Table 1.)

**Table 1:** Age of Patients

Age of the patient	Frequency %
18–29	29.7%
30–39	26.2%
40–59	40.9%
60+	3.2%

The participants in the age range of 40–59 years old accounted for almost 41% of all participants. Other patient participants were around 18–29 years of age (29.7%) and 30–39 years (26.2%) and 3.17% of them were over 60 years of age. More females (74.3%) were accounted in the group of subjects when compared to males which were 25.07% in number and 2 others did not wish to disclose their gender.

When looking at the level of education, High school accounted for around 50% out of all the participants, followed by around 18% who had a Bachelor’s degree and 14% who had an associate degree (Table 2.)

**Table 2:** Patients’ level of education

Level of Education	Frequency%
Elementary	8.7%
High school	**50.1%**
Associate’s degree	14.6%
Bachelor’s degree	18.2%
Masters or doctorate	8.4%

Almost 59% of the patients took 40 min or less while 28% took more than 40 min to an hour to get to orthodontic in-person appointments at UIC COD orthodontic clinic. On the other hand, around 10% took 60–89 min to travel.

Around 46% agreed that they had used the Teleorthodontic application more than once which may be due to the fact that more than 90% of the patients found it easy and simple to use this Teleorthodontics application.

**Table 3:** Reasons for using this Teleorthodontic application by patients

For what reason did you use this Teleorthodontic application?	Frequency%
New patient consultation	20%
Follow up regarding orthodontic treatment	50%
Emergency	5%
COVID-19 related issues	3%
Other	5%
Emergency + Other	0.3%
COVID + Other	0.6%
New patient consult + Other	0.6%
New patient consult + COVID-19 related + Other	0.3%
New patient consult + Emergency	0.3%

The majority of patients (50%) used this application for evaluation of treatment progress, retainer checks, and treatment consultation while 20% of the participants used this application for new patient consultation. Only 5% were emergencies, 3% were COVID-19 related and the rest of the reasons varied from broken brackets, and loose wires to cancellations or any changes in appointments which was around 9.3%. (Shown in Table 3.)

**Graph 1:**
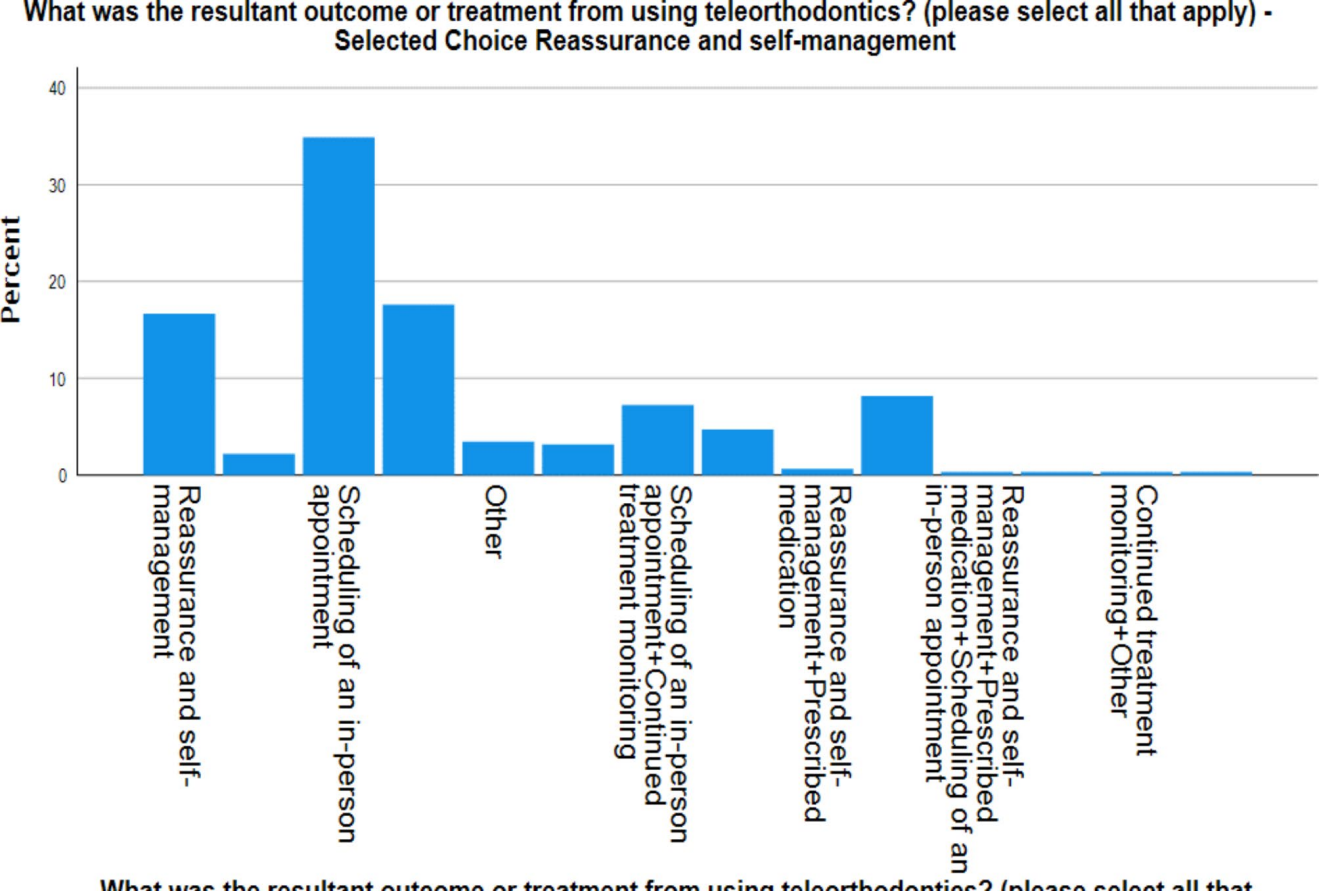
Resultant outcome from patients using the Teleorthodontic application

The resultant outcome (Graph 1) by using the Teleorthodontics application was scheduling an in-person appointment (41.4%), 27.4% was continued treatment monitoring and 25.3% was reassurance and self-management. Only 2.7% was for prescribed medication.

Most patients preferred using SMS text messaging which was around 47.17% followed by 34.35% who used Phone (voice only) and 10.87% who used email and 3.7% of them used other modes like Video conference, Zoom, Webex, etc. DM was used only by 1.3% Around 1.74% of them sent pictures or did not use any mode of communication.

When asked if the Teleorthodontic application helps them feel more personally connected to their orthodontic providers, 54.14% of the patient participants strongly agreed, 33.44% agreed, 6.69% somewhat agreed and 5.10% were neutral – neither agreed nor disagreed.

**Graph 2:**
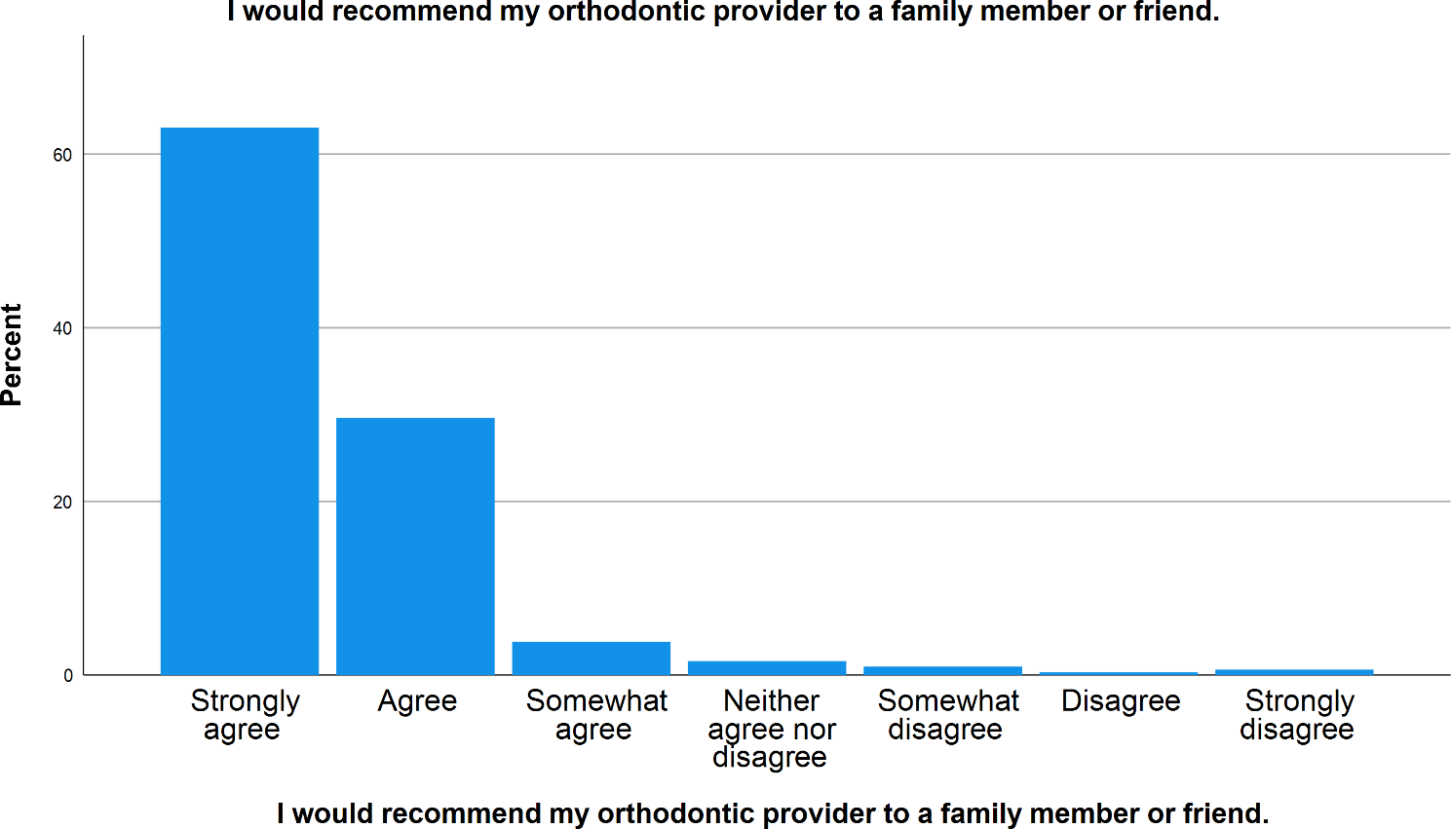
Recommendation of the application to family and friends

Graph 2. shows that the vast majority of patients (95%) agreed when asked if they would recommend their orthodontic providers to family and friends, (63% strongly agreed, 29% agreed and 3% somewhat agreed) which was probably because of the fact that 95% of them agreed that they understood the orthodontic provider’s responses clearly through Teleorthodontics (54% strongly agreed, 38% agreed and 3% somewhat agreed as shown in Table 4.)

**Table 4:** Understanding the orthodontists’s response through the Teleorthodontic application

I could understand my orthodontic provider’s responses clearly through this Teleorthodontic application.	Frequency%
Strongly agree	54.8%
Agree	38.1%
Somewhat agree	3.2%
Neither agree nor disagree	2.2%
Somewhat disagree	1.0%
Strongly Disagree	0.6%

**Graph 3:**
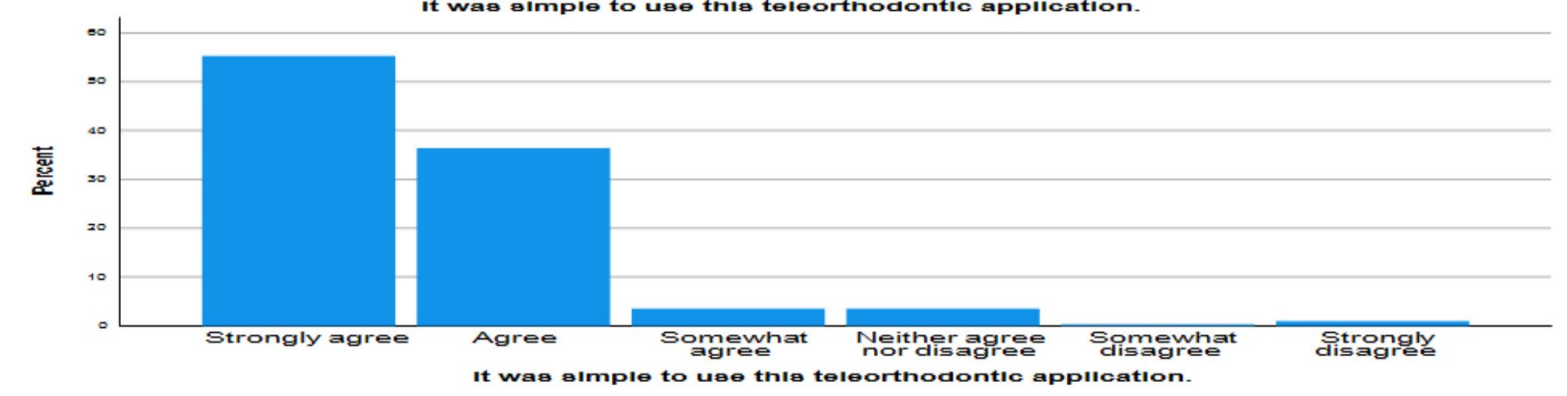
Ease of use of the application by patients

94% of them agreed that they found the Teleorthodontic application simple and easy (Graph 3.) to use and understand and also found the interaction with the application pleasant.

93% of the patients agreed that they could become productive quickly using this application and around 92% agreed that they liked using this application.

94% agreed that they found the application simple and easy to understand and 92% of them agreed that they could express themselves effectively through this application too.

When asked whether the interaction was the same as in-person visits, around 79% of them agreed and the rest around 11% of them disagreed while 7% neither agreed nor disagreed. (Table 5.)

**Table 5:** Showing the percentage of agreement/disagreement if the application was the same as in-person visits

I think my interaction with my orthodontic provider through this tele orthodontic application is the same as in-person visits.	Frequency%
Strongly agree	29.5%
Agree	30.8%
Somewhat agree	20.1%
Neither agree nor disagree	7.1%
Somewhat disagree	4.5%
Disagree	5.5%
Strongly disagree	2.3%

The agreement in involvement in orthodontic care and treatment in using the Teleorthodontic application was around 86%.

Around 66% of them said that this tele application saved them time in traveling to the orthodontic clinic, 32.4% strongly agreed and only around 8% disagreed with this.

**Graph 4:**
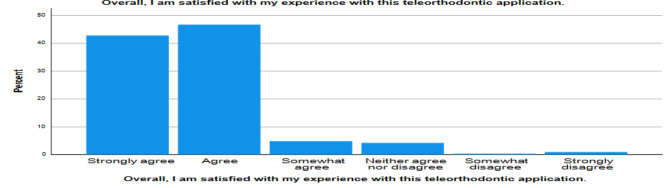
Overall satisfaction of the patient with the different forms of Teleorthodontic applications

Graph 4. Shows that 92% of the patients agreed that they would use the application again and overall satisfaction with the experience was also 92% which shows that the Teleorthodontic application can be used regularly as an adjunct to normal orthodontic, in-person appointments.

As for the providers, around 28% were orthodontic residents, while almost 39% were orthodontic faculty members (Table 6), 22% were below the age of 29 years, while others were 30–39 years (16.7%), 40–59 (16.7%), and 60+ (8.3%). Contrary to the patients, here males were more in number (36%) when compared to females (27%).

**Table 6:** The percentage of Orthodontists/Providers that used Teleorthodontics

Orthodontist / Provider	Percentage
Faculty member	27.8%
Orthodontic Resident	38.9%

33% agreed that they felt more personally connected to their patients and around 16% strongly agreed (Table 7). 19% agreed that the interaction with their patients through the use of the application was the same as in-person visits and around 11% strongly agreed.

**Table 7:** Percentage of feeling personally connected to the patient through this application

	Frequency %
Strongly Agree	16.7%
Agree	33.3%
Neither agree or Disagree	2.8%
Disagree	5.6%

Similar to the patients, the orthodontists (around 22%) also agreed that the application saved them time traveling and 19% strongly agreed. Most of the faculty and residents (41%) said they took 40 min or less, while almost 14% took between 40 min and an hour. The rest (5.6%) took 90 min or more to travel to the UIC College of Dentistry.

It was interesting to note that 25% of them preferred using the video conferencing application when compared to 22% who preferred SMS texting on the phone.

66% of them had high-speed internet available at home and 47% of them used Teleorthodontics more than once and 36% agreed that they had a pleasant interaction with the orthodontic application. 22% strongly agreed that it was simple to use and 19% agreed. While 30% agreed it was easy to communicate with the patient with the help of this application 22% strongly agreed.

Almost 28% of them agreed that they felt comfortable using this Teleorthodontic application to interact with their orthodontic patients while 19% strongly agreed.

Also, 30% of them agreed that they would use this application again and 33% agreed that they would recommend this to friends and family and 13% strongly agreed too.

When asked if it would be beneficial to incorporate training on Teleorthodontic application services in the curriculum, 25% of the orthodontists agreed, while 13% strongly agreed.

When looking at overall satisfaction, 30% agreed that they were satisfied with this application while 16% strongly agreed.

On the whole, it is good to note that the Teleorthodontic application was extremely useful to many patients and was a simple and easy method of communication to receive good quality orthodontic care, especially during the peak of the COVID-19 outbreak.

## Discussion

The outbreak of COVID-19 has presented numerous challenges for oral healthcare. Dental care professionals often work in close proximity to patients, making infection control a top priority. Preventing the spread of infection, particularly through aerosols, is crucial to safeguarding the health of both patients and dental care professionals. As a result, many healthcare providers have implemented new protocols and safety measures, such as increased use of personal protective equipment, modifications to ventilation systems, and virtual consultations, to reduce the risk of transmission [5, 6].

Due to limited access to dental clinics and hospitals during the lockdown period, life became particularly difficult for people living in rural areas who relied on city hospitals and clinics for healthcare services. This issue was especially challenging in emergency situations [7].

Teledentistry is a wonderful tool that has helped many patients receive simple and effective dental consultations and treatment advice in certain cases [8]. One of the most common uses of teledentistry is to receive electronic prescriptions for infections, inflammation, or pain, and schedule follow-up appointments [9].

Teleorthodontics has revolutionized the way orthodontic care is provided to patients. By offering safe and convenient treatment options, patients no longer have to visit a dental office and risk exposure to COVID-19. With it, patients can save time by not needing to take time off work or school to visit an orthodontist, and orthodontists can treat more patients in a shorter amount of time. We implemented Teleorthodontics to test the quality of care we could provide. Based on the feedback received from patients and parents/guardians of patients through questionnaires, we were able to gain insight into the effectiveness of the application for dental care.

It is important to note here that this application was used as just an adjunct or addition and not a replacement for regular in-person dental appointments. This was introduced mainly during the COVID-19 outbreak when all clinics were closed due to lockdown [10].

After reviewing the survey results, it appears that people were quite receptive to Teleorthodontics. Respondents found the tool to be user-friendly and straightforward, leading to high levels of satisfaction. Many participants indicated that they would be willing to continue using the technology in the future. Additionally, most individuals reported that they found the experience to be comfortable and enjoyable, despite the lack of in-person appointments. While the personal touch of in-person consults was missed, many patients still found the remote consultations to be informative and satisfactory. Many patients reported that their problems and concerns were addressed through virtual appointments, eliminating the need for in-person visits. Traveling to appointments can be a hassle, as it often involves long drives and waiting times at the clinic that can be exhausting. Additionally, the time and money spent on travel can be a burden for many. Whereas Teleorthodontics is incredibly easy, precise and convenient that many participants have reported they would even recommend this type of dental care to their loved ones. Not only does it save time, it also reduces fuel consumption, making it an eco-friendly option that helps reduce the number of harmful gas emissions in the atmosphere.

Teledentistry has been widely accepted by patients and has shown promising results in various studies. For instance, *Ruiz-Lopez del Prado et al*. conducted a study using questionnaires to evaluate the state of oral health in preoperative anesthetic evaluations, and found that patients responded positively to the use of teledentistry. This suggests that teledentistry could be a valuable tool in oral healthcare delivery [11].

*Uribe et al* used a questionnaire to evaluate patients’, parents’, and orthodontists’ perspectives on orthodontic treatment duration and techniques on the rate of tooth movement [12].

Our study builds upon the work of *Dalessandri et al*. who examined the acceptance and efficacy of Dental Monitoring among patients and orthodontists. While their study used questionnaires to assess attitudes towards the tool, we went a step further by incorporating additional modes of communication alongside direct messaging. Overall, like *Dalessandri et al.* we found a positive outcome in terms of patients’ and dentists’ acceptance. *Saccomanno et al.* recently published a review of 8 articles between March 2020 to March 2021 vs. December 2019 to November 2021 showing mixed reviews on teledentistry [13] *Caprioglio et al.* treated orthodontic emergencies via Teleorthodontics (email, phone call, Skype, or Zoom), by gathering information initially about the patient’s health status and then guided the patients to resolve their emergencies [14].

*Arqub et al.*, found that phone call or message was preferred by 73% of patients, avoiding visits in person during the pandemic which was very similar to our study where 47% of them were comfortable and preferred using SMS text messaging when compared to coming to the clinic [15].

In the study conducted by *Cotrin et al.*, questionnaires were used, which were completed by orthodontists from four different Brazilian universities. The study looked into the most common orthodontic emergencies and the tool that was most often required by the orthodontist via Teleorthodontics, in this case, which was a WhatsApp/telephone call [16].

Another study conducted by *Saccomanno S. et al*. in 2020 in Italy showed the advantage of Teleorthodontics which has become increasingly popular, especially during the pandemic lockdown, as it allows for more efficient troubleshooting and follow-up of cases [17]. The various modes of Teleorthodontics, such as video-calls, text messaging, and photo sharing, have proven to be effective in providing orthodontic care remotely. A recent survey study evaluated the impact of COVID-19 on orthodontic practice management and found that approximately 52% of orthodontic providers offered teledentistry in their practice after the pandemic. This suggests that Teleorthodontics is a promising solution and is likely here to stay [18–21].

From our study, it is clear that Teleorthodontics is highly beneficial not only during crises like the COVID-19 pandemic, but also for everyday use such as follow-up appointments, tracking oral hygiene, and addressing minor concerns. While Teleorthodontics does come with certain limitations, such as technical difficulties and the inability to conduct physical examinations, there are also numerous advantages, such as the convenience of avoiding travel time and minimizing the risk of infection transmission by reducing the time spent waiting in a clinic [22–29].

Communicating with the orthodontist and resolving issues from anywhere and at any time is a game-changer for patients [30, 31]. Whether it’s during the period of a lockdown or a regular follow-up visit, Teleorthodontics provides a sense of relief. Teleorthodontics is not a replacement for regular in-office visits, but it is an excellent complement to traditional orthodontic care. It improves dental care accessibility and is more cost-effective. A limitation of the study is that the sample is drawn from one institution. Consequently, the results may not be generalizable. The study findings should be interpreted keeping this limitation in perspective.

## Conclusion

Teleorthodontics has benefited both patients and orthodontists. It offers several advantages, including a reduced risk of infection transmission. In traditional dental clinics, infection control is always a concern. With Teleorthodontics, the risk is significantly lower, making it a safe and convenient option for patients. Based on our survey results, we highly recommend incorporating Teleorthodontics into regular department services, particularly for follow-up or recall appointments since it is an excellent way to enhance patient care and improve access to dental services.

## Data Availability

The datasets used and/or analyzed during the current study are available from the corresponding author upon reasonable request and are included in this published article.
